# Book Review: Practical Guide to Medical Student Assessment

**DOI:** 10.3352/jeehp.2006.3.6

**Published:** 2006-12-18

**Authors:** Sun Huh

**Affiliations:** Department of Parasitology, College of Medicine and Institute of Medical Education Hallym University, Chuncheon, Korea.

This tiny book surprised me with its brief, lucid description of the assessment tools used in medical schools. The authors call it a practical reference to commonly raised questions about assessment instruments, and it is a good guide for medical teachers like me. It deals with most of the assessment tools for medical education with which I am acquainted, and it is very suitable for medical teachers who are not specialists in education. After less than a day of reading, I could swim the sea of medical student assessment. The book consists of four sections: Principles and Purpose of Assessment; Assessment of "Knows" and "Knows How"; Assessment of "Shows How"; Assessment of "Does."

Section 1 emphasizes the key concept that "students were derived by assessment." My students' greatest concern is examinations. Students value passing exams and getting higher marks, since teachers, including me, usually evaluate students based on their credit records. Therefore, the validity and reliability of the assessment tools are the most critical considerations for teachers. These are especially important in high-stake tests, such as the licensing examination. The utility of an assessment instrument is based on a careful consideration of several factors, including its reliability, validity, educational impact, cost, and accessibility. The authors describe this key concept well. An assessment in clinical medicine should consider special issues, since a difference in conditions affects student performance in a manner unlike paper and pencil examinations. To increase the level of generalizability, that is, the reproducibility of measurement, multiple sampling across different skills and domains is required. In addition, the norm-referenced standard and criterion-referenced standard are explained. The last part of Section 1 describes Miller's Pyramid, which outlines clinical competence at multiple levels. The assessment levels and corresponding examples are displayed based on the "Knows," "Knows How," "Shows How," and "Does" pyramid. For example, "Shows How" matches the "Objective Structured Clinical Examination (OSCE), Long Case and Short Case." The box "Recommendations for Better Practices" briefly describes the core knowledge for teachers. It is a very useful design, since the contents of the box can be used during teacher training workshops.

Section 2 presents various methods for assessing "Knows" and "Knows How," with recommended situations and the effect of each method, including oral examinations, long essay questions, short answer questions, multiple choice questions, extended matching items, and the key feature test. My school does not use the key feature test, which was originally developed by the Medical Council of Canada for its licensing examination. It is an interesting test, since it is a clinical scenario presented as a paper and pencil examination that can be adapted for student assessment in medical schools. The authors explain each assessment method clearly and provide an example.

Section 3 is for assessing "Shows How." It deals with long and short cases involving real patients, as well as the OSCE. Since the OSCE is the method commonly used for assessing clinical performance, the outline of the method and example presented in this section will be very helpful for medical instructors.

The last section introduces the assessment of "Does" as a mini-clinical evaluation exercise, direct observation of procedural skills, clinical work sampling, ward checklist, 360-degree evaluation, logbook, and portfolio. Again, the last three methods are not used in my school, and it will be difficult for one school to adopt all of these assessment methods. However, the various methods can be used in individual departments or hospitals and the results can be compared using the correlations among the methods.

This publication is not a handbook, but a synopsis. Therefore, its contents should be a useful guide in workshops for new faculty or on various educational topics in medical school. The authors targeted the needs in the field of medical education accurately. Furthermore, the contents of this book may be applicable to other fields of education, such as dentistry or nursing, since those disciplines also involve a clinical setting. The book is very well edited, and I found no typographical errors or obscure explanations. I was able to summarize the student assessment tools while reading the book.

Two of the coauthors have previously published a book titled "Basics in Medical Education," [1] which also contained assessment and evaluation sections describing a road map to student assessment, multiple choice questions, easy questions, oral examinations, standardized patients, and portfolios. The new book approaches assessment from the perspective of a practical guideline for medical teachers, and I believe that it is a good hand-held guide.
